# Novel chytrid lineages dominate fungal sequences in diverse marine and freshwater habitats

**DOI:** 10.1038/srep30120

**Published:** 2016-07-22

**Authors:** André M. Comeau, Warwick F. Vincent, Louis Bernier, Connie Lovejoy

**Affiliations:** 1 Institut de Biologie Intégrative et des Systèmes (IBIS) and Centre d’Étude de la Forêt (CEF), Université Laval, Québec, Canada; 2 Centre d’Études Nordiques (CEN), Takuvik Joint International Laboratory (CNRS UMI-3376) and Département de Biologie, Université Laval, Québec, Canada; 3 Institut de Biologie Intégrative et des Systèmes (IBIS), Takuvik Joint International Laboratory (CNRS UMI-3376) and Département de Biologie, Université Laval, Québec, Canada

## Abstract

In aquatic environments, fungal communities remain little studied despite their taxonomic and functional diversity. To extend the ecological coverage of this group, we conducted an in-depth analysis of fungal sequences within our collection of 3.6 million V4 18S rRNA pyrosequences originating from 319 individual marine (including sea-ice) and freshwater samples from libraries generated within diverse projects studying Arctic and temperate biomes in the past decade. Among the ~1.7 million post-filtered reads of highest taxonomic and phylogenetic quality, 23,263 fungal sequences were identified. The overall mean proportion was 1.35%, but with large variability; for example, from 0.01 to 59% of total sequences for Arctic seawater samples. Almost all sample types were dominated by Chytridiomycota-like sequences, followed by moderate-to-minor contributions of Ascomycota, Cryptomycota and Basidiomycota. Species and/or strain richness was high, with many novel sequences and high niche separation. The affinity of the most common reads to phytoplankton parasites suggests that aquatic fungi deserve renewed attention for their role in algal succession and carbon cycling.

High-throughput sequencing methods have augmented our capacity to assess microbial eukaryotic diversity and related function in microbial ecology. In freshwater and marine environments, attention has often been placed on protist diversity including phytoplankton and heterotrophic flagellates^1^,^2^, but the fungal components are often set aside or given cursory analyses without considering finer taxonomic levels. This contrasts with microbial studies on soil environments, in which fungi are considered the primary agents of decomposition^3^. The lesser attention to aquatic fungi is perhaps due to their overall low abundances in marine clone libraries (~1% of total eukaryotes^4^) and a perception that they may therefore be of little ecological relevance. However, some studies have shown larger proportions of fungi^5^ and the taxonomic richness of fungi in marine environments is reported to be high^6^. One group especially, the Chytridiomycota, has been identified as an early divergent lineage implicated in a variety of ecological processes. The role of Chytridiomycota as parasites of freshwater algae is well known^7^,^8^ and taxa in this group are thought to mediate the transfer of organic matter from phytoplankton into zooplankton via saprophytic and parasitic activity described as the “mycoloop”^9^. Freshwater chytrids are also known as parasites of zooplankton^10^ and of larger animals such as amphibians^11^. However, Chytridiomycota are rarely reported from marine environments and there are few marine cultured isolates^12^. Based on studies to date, it has been suggested that Dikarya (Ascomycota and Basidiomycota) dominate marine fungi^4^,^13^ and hyphomycetes (mostly assigned to the Ascomycota) play a major role decomposing leaf litter and other terrigenous substrates in rivers and streams^14^.

Direct examination of fungal diversity using high-throughput sequencing has been increasingly applied^13^,^14^,^15^,^16^, but data are still lacking in many environments, especially pelagic and polar regions. To more broadly assess aquatic fungal diversity, we examined pyrosequencing libraries of the V4 region of 18S rRNA (DNA and RNA) generated within our research group’s 19 projects studying Arctic and temperate biomes in the past decade (Fig. 1). The database totaled more than 3.6 million sequences, originating from 319 individual marine, freshwater and sea-ice samples (0.2–50 μm size-fractions), from 103 locations sampled from 2003 to 2011 (Supplementary Table S1).

## Results and Discussion

Our analysis showed the presence of fungi in all samples tested. The initial raw reads were reduced to ~1.7 million reads of highest taxonomic and phylogenetic quality using the filtering methods outlined below. Among these, 23,263 fungal sequences were identified, for an overall rate of incidence of 1.35%.

**Figure 2:**
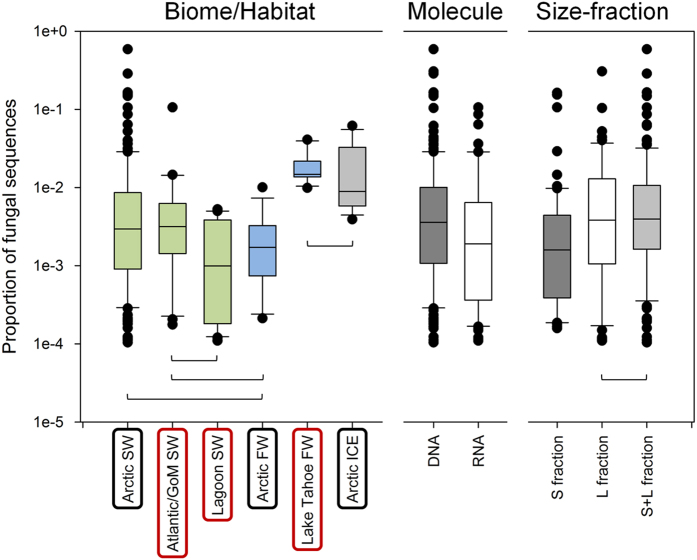
Incidences of fungal sequences. Box-plots of proportions of fungal sequences in the different biomes/habitats (left panel), in DNA vs. RNA samples (middle panel) and in the different size fractions (right panel) – small (S), large (L) or when both were mixed (S + L). Boxes are significantly different from all other boxes in the same panels (Kruskal-Wallis [Bonferroni corrected] or Mann-Whitney, p < 0.05), unless linked by brackets. FW, freshwater; SW, seawater.

There were large variations among and within habitat types (Fig. 2), but the overall mean incidences were uniformly <5%. For example, there was large variability (0.01–59%) in Arctic seawater, which represented the greatest number of samples and overlapped with the ranges of other seawater habitats. There were, however, significantly higher proportions of fungi in sequences from temperate freshwater and Arctic sea-ice compared to the other habitats. There were also slightly higher fungal proportions in DNA relative to RNA (cDNA) libraries, possibly because resting stages, including spores, may have been more numerous among the fungal reads, leading to higher numbers in DNA signals. The higher proportion of fungal reads in the DNA derived libraries could also reflect the preservation of non-living DNA in cold waters^17^,^18^. Higher proportions of fungi occurred in the large size-fractions (>3 μm) that, in part, may reflect the physical association with detritus and nanoplankton. There were no significant correlations between the different fungal taxa and month, season, depth, temperature or salinity. However, there was a slight negative correlation between total fungi and temperature within the temperate samples, which were from sites spanning the largest temperature range, suggesting a preference for warmer water column conditions (Supplementary Fig. S1).

A total of 44 genera (plus one clone and one informal genus) of fungi were identified, contained within 9 (sub)phyla (including *incertae sedis*), a testament to the large richness of fungi that can be captured by the universal 18S primers. Although ITS regions are often used to examine species- and strain-level fungal diversity, 18S rRNA sequences have proven valuable in many fungal analyses^13^,^19^,^20^,^21^,^22^. In the present study, our moderately-sized (~440 bp) V4 amplicons resolved fungal sequences to at least the genus level, confirmed by manual BLASTn of taxonomic identifications. The full taxonomic breakdown of the samples is presented in Supplementary Table S2.

**Figure 3:**
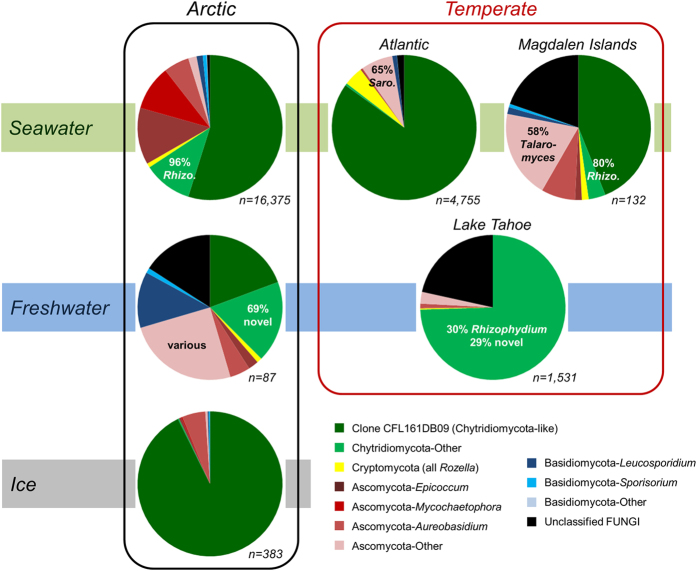
Taxonomic identities of fungal sequences in the various biomes/habitats. The total numbers of sequences (*n*) in each category are indicated. Rhizo., *Rhizophydium*; Saro., *Sarocladium*.

A striking overall feature of our fungal sequence dataset was that almost all sample types were dominated by Chytridiomycota-like sequences (38–93% for the groupings in Fig. 3). This was followed by moderate contributions of Ascomycota (4–32%) and relatively minor contributions of Cryptomycota (0–4%; all *Rozella* spp.) and Basidiomycota (0–14%). For the Basidiomycota, *Leucosporidium* was the most common genus (notably in Arctic freshwaters), whereas within the Ascomycota, *Epicoccum*, *Mycochaetophora*, *Aureobasidium*, *Talaromyces* and *Sarocladium* were recovered in varying proportions. The high dominance of chytrids in Arctic sea-ice (93%) agrees with a recent 18S V2-V3 Alaskan study showing 70–95% chytrids among fungal sequences from land-fast ice and underlying marine sediments^22^. In contrast, a recent meta-analysis by Tisthammer *et al.*^13^ focused on marine water and sediments found that Dikarya were dominant and chytrids were relatively rare. However, their study was based upon only 56 samples from 33 sites, identified less than half the number of fungal sequences as our study, and had a limited coverage of polar regions. They also targeted the small ~65 bp V9 variable region of the 18S rRNA gene and, consequently, greater than 50% of their 10,793 sequences remained unidentified. Our V4 analysis with a larger dataset over a broad range of aquatic environments, with emphasis on planktonic and sea-ice systems, implies that chytrids may be more abundant than previously suspected.

**Figure 4:**
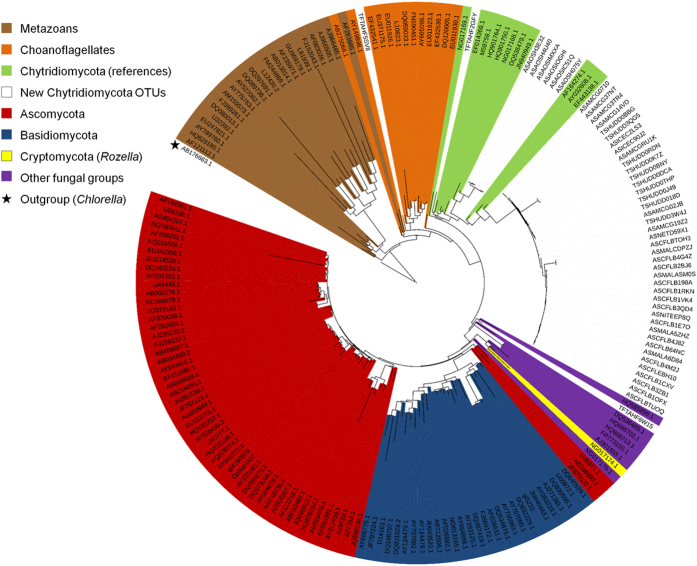
Phylogeny of the top 50 Chytridiomycota-like OTUs (98% identity) from all samples along with reference sequences. This 18S maximum-likelihood tree of the V4 region only ( = 401–462 bp) was obtained with RAxML (with 100 bootstrap replicates) and rooted with a *Chlorella* 18S sequence (Viridiplantae; AB176663.1). Fungi color-coding matches Fig. 3. Reference sequences are identified by their NCBI accession numbers and novel sequences from this study are identified as follows: (A/T) + (F/S) + project + uniqueID; where A/T is Arctic vs. Temperate, F/S is Freshwater vs. Seawater, the project is identified with a 3-letter code (AMC = AMCE, AOS, CFL, HUD = Hudson, ICE = ICESCAPES, MAL = MALINA, NET = ArcticNet’11, NIT = ANITA, TAH = Lake Tahoe; Supplementary Table S1), and a 5-letter code identifies the representative sequence for the OTU (allowing for retrieval in Supplementary File S1).

Within the Chytridiomycota, sequences with closest match to uncultured clone CFL161DB09 (HM561158.1) were by far dominant, except in Lake Tahoe where it was clone PA2009D12 (HQ191408) from meromictic Lake Pavin^15^, followed by *Rhizophydium*-like sequences. Clone CFL161DB09 originates from an Arctic seawater sample from the Amundsen Gulf and is representative of a small cluster of clones from the same study^23^. These Arctic clones, and the large set of additional sequences from this study, group together with known Chytridiomycota at the base of the fungal phylogenetic tree (Fig. 4). However, the closest cultured species, *Clydaea vesicula* (alternatively annotated as “Chytridiales sp.” JEL369 [EF443137] and PL70 [EF443138]) within the Lobulomycetales^24^, shares only ~91% identity, therefore these environmental sequences represent novel lineages of aquatic Chytridiomycota.

To assess the overall diversity of the fungal sequences, they were clustered into Operational Taxonomic Units (OTUs) at 98% identity and a shared OTU analysis was conducted between all sample type combinations (latitude vs. source medium; Supplementary Fig. S2). We used the clustering technique as a tool allowing for these β-diversity comparisons between samples, however we add the caveat that the 98% level may not be strictly appropriate for all fungal groups; some may require higher identity, some less, to reflect accurate species-level clusters. Nonetheless, little overlap was observed between freshwater, seawater and sea-ice within the same biomes, indicating that species (or strain) richness was high and that environmental niche separation was strong. Only 168 OTUs (representing ~4% of the total set) were universally shared between the polar and temperate latitudes, and the majority of these were identified as CFL161DB09-like novel Chytridiomycota (Supplementary Fig. S3). Although shared, an examination of the distributions of the top 40 dominant OTUs (Supplementary Fig. S4) showed inverse sequence count patterns between the two biomes, indicating that each environment and aquatic medium was populated by its own distinct lineages or strains. Similar situations were recently encountered for land-based Chytridiomycota full-length 18S clones from high-elevation soils and snow^19^,^20^. Comparison of soil and aquatic Chytridiomycota was outside the scope of the present study, but such analysis in the future would yield interesting insights into the evolution of this group. High niche or biome compartmentalization was also observed in a meta-analysis of a small set of full-length 18S fungal sequences (~1,800) by Panzer *et al.*^21^ using coarse habitat definitions (e.g., all seawater samples grouped as one) and the Tisthammer *et al.*^13^ meta-analysis comparing pelagic and benthic communities.

The ecological roles of the novel lineages detected in the present study are unknown, however many chytrids, including numerous species within the genus *Rhizophydium,* have long been known as algal parasites^7^. Lepère *et al.*^25^ recently demonstrated direct *in situ* hybridization evidence, albeit relatively uncommon, of fungi associated (potentially parasitically) with marine photosynthetic picoeukaryotes using specific chytrid and general fungal probes. In our study, significant positive correlations (Supplementary Fig. S1) were found between the proportions of Chytridiomycota-like sequences and either total chlorophyll *a* concentrations or proportions of diatom sequences, albeit with an r_s_ of 0.29. More detailed studies following species-specific blooms over time using quantitative PCR or fluorescence *in situ* hybridization techniques are clearly needed. This broad meta-analysis of aquatic fungal sequences highlights the importance of further examining the roles of Chytridiomycota in marine and freshwater environments through molecular methods, and the need to pursue cultivation of dominant lineages such as arctic clone CFL161DB09 for use in physiological and ecological studies.

## Materials and Methods

### Sample collection and pyrosequencing

Sample collection, nucleic acid extractions and pyrosequencing are described in associated publications (listed in Supplementary Table S1) and were similar for all data sets, including those listed as unpublished from our research group. Briefly, to ensure sufficient biomass collection without blocking filters, 1–10 L of seawater or freshwater, and 0.2–0.5 L of melted ice, were filtered sequentially into small (S = 0.2–3 μm) and large (L = >3 μm) size-fractions. DNA was extracted using a modified salt-based method and RNA was extracted using the RNeasy Mini Kit (Qiagen), including a DNase treatment and followed immediately by cDNA synthesis, as described in Charvet *et al.*^26^ (excluding the MDA step). The V4 region of the 18S rRNAs (RNA) and rRNA genes (DNA) were amplified, pooled and pyrosequenced as described in Comeau *et al.*^27^,^28^. For some samples and stations only one molecule (DNA or RNA) or one size-fraction (L or S) was sequenced, but for many both were done in parallel. In some cases, the size-fractions were either mixed 50:50 or in accordance with the corresponding chlorophyll *a* or cell count ratios measured in each fraction (Supplementary Table S1).

### Read quality screening and selection for fungi

Quality filtering (and further analyses herein) was conducted using mothur ( www.mothur.org). The 319 samples contained a cumulative 3,651,419 raw reads which were quality filtered as described in Comeau *et al.*^27^,^28^, but with a more stringent requirement of a 400 bp minimum length in order to only retain reads of highest taxonomic utility and phylogenetic quality for this heavily identification-based study. This resulted in 1,717,227 filtered reads: 1,032,555 Arctic marine, 53,456 Arctic freshwater, 21,829 Arctic ice, 527,710 temperate marine and 81,677 temperate freshwater. Reads were then screened using a custom 18S database (see below) at the “phylum” (eukaryote “major group”) level to select fungal sequences. Cumulatively, 23,263 fungal sequences were retained among all samples.

### OTU generation and taxonomic identification

The fungal sequences were merged, aligned and OTUs were formed at the 98% similarity level (furthest-neighbor method; Supplementary File S1) following the recommendation for using a maximum of 98% with 454 GS-FLX Titanium chemistry to avoid misidentifying V4 Eukarya tags^29^. This permitted construction of rank-abundance curves for each sample type (α-diversity; Supplementary Fig. S4) and enabled a shared OTU analysis to examine sample type overlap (β-diversity; Supplementary Figs S2 and S3). Final taxonomic identification of OTUs and sequences was conducted using an 18S database developed in-house, originally described in Comeau *et al.*^27^,^28^, but updated to be more inclusive of fungal diversity, including uncultured environmental clones^30^. This database was also further refined by an iterative process whereby major “unclassified” fungal sequences from this study were manually investigated by BLASTn in GenBank nr (blast.ncbi.nlm.nih.gov), new reference sequences were added (if found), and the classification process for all sequences was repeated until satisfactory identification was achieved.

### Phylogenetic analysis

The top 50 Chytridiomycota-like OTUs were aligned with other fungal and metazoan reference sequences from GenBank, trimmed to only the V4 region (401–462 bp), using the implementation of ClustalW in BioEdit ( www.mbio.ncsu.edu/bioedit/bioedit.html). The eye-refined alignment was used to construct a maximum-likelihood tree in RAxML (sco.h-its.org/exelixis/web/software/raxml/) with 100 bootstrap replicates, rooted with a *Chlorella* sequence (Viridiplantae; AB176663.1). The tree was visualized and exported using the Interactive Tree of Life (iTOL; itol.embl.de).

### Statistical analysis

Non-parametric (non-normal) Mann-Whitney and Kruskal-Wallis tests were used to verify differences between groups and Spearman’s *r* (on log-transformed data) was used for correlations. F- and t-tests (equal or unequal variance, as was the case) and linear regressions (log-transformed data) were used with data that fulfilled the conditions of normality. All statistical analyses were carried out with PAST (folk.uio.no/ohammer/past/).

## Additional Information

**How to cite this article**: Comeau, André M. *et al.* Novel chytrid lineages dominate fungal sequences in diverse marine and freshwater habitats. *Sci. Rep.* **6**, 30120; doi: 10.1038/srep30120 (2016).

## Supplementary Material

Supplementary Dataset 1

Supplementary Figures

Supplementary Tables

## Figures and Tables

**Figure 1:**
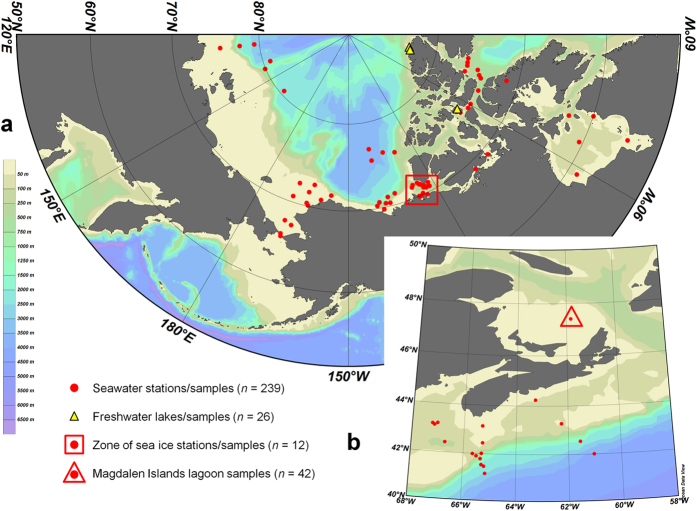
Locations of sampling sites included in pyrosequencing libraries. (**a**) Arctic samples, ranging through Canadian, American and Russian territories. The box outlines the area of origin for the sea ice samples and the bathymetric scale at left applies to both maps. (**b**) Temperate samples from the Atlantic Ocean, including the Magdalen Islands lagoon samples (triangle). Not shown is the location of the Lake Tahoe samples (39°06’N 120°02’W). Maps created with Ocean Data View version 4 ( https://odv.awi.de/en/home/).
