# Supplementary material for: Novel chytrid lineages dominate fungal sequences in diverse marine and freshwater habitats
Source: Sci Rep. 2016 Jul 22;6:30120. doi: 10.1038/srep30120 (PMC4957111; doi:10.1038/srep30120)

Supplementary Information for:

Novel chytrid lineages dominate fungal sequences in diverse marine and freshwater habitats

André M. Comeau, Warwick F. Vincent, Louis Bernier & Connie Lovejoy

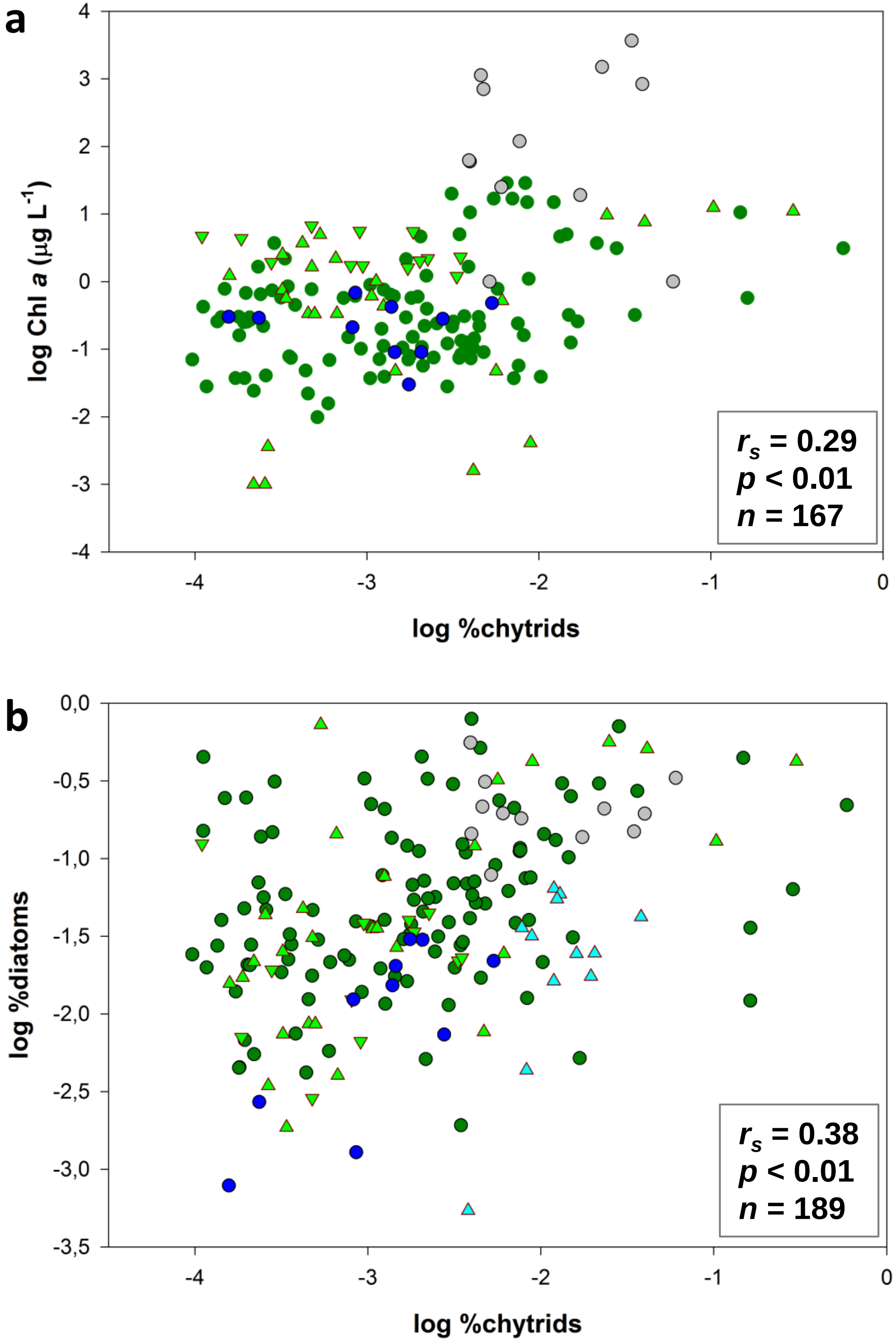

Figure S1. Biotic and abiotic parameters correlated to proportions of total fungi or Chytridiomycota-like sequences. Weak, but significant, positive correlations are present between proportions of chytrids and either chlorophyll *a* (**a**) or proportions of diatoms (**b**) for all sample types together. Note that data on chlorophyll *a* was not available for Lake Tahoe. A slightly stronger, but negative, correlation was seen between total fungi in temperate locations only and temperature (**c**). Listed are Spearman's *r* values along with the *p* values and sample sizes (*n*) for each plot. FW, freshwater; SW, seawater.

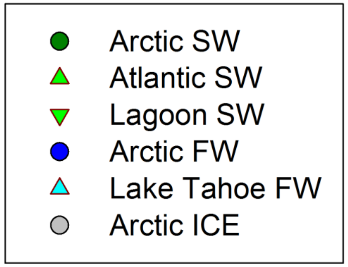

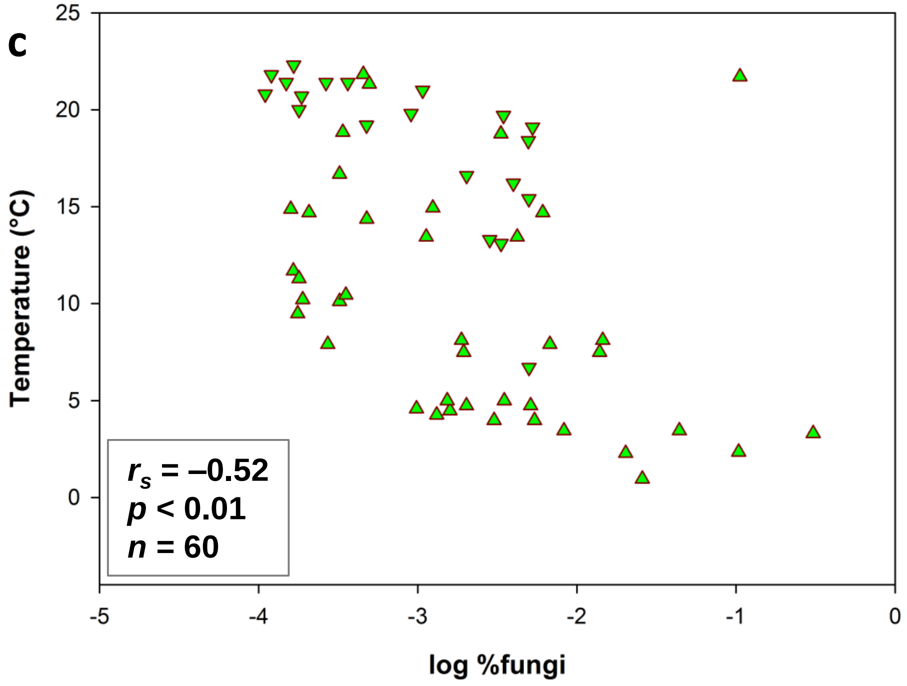

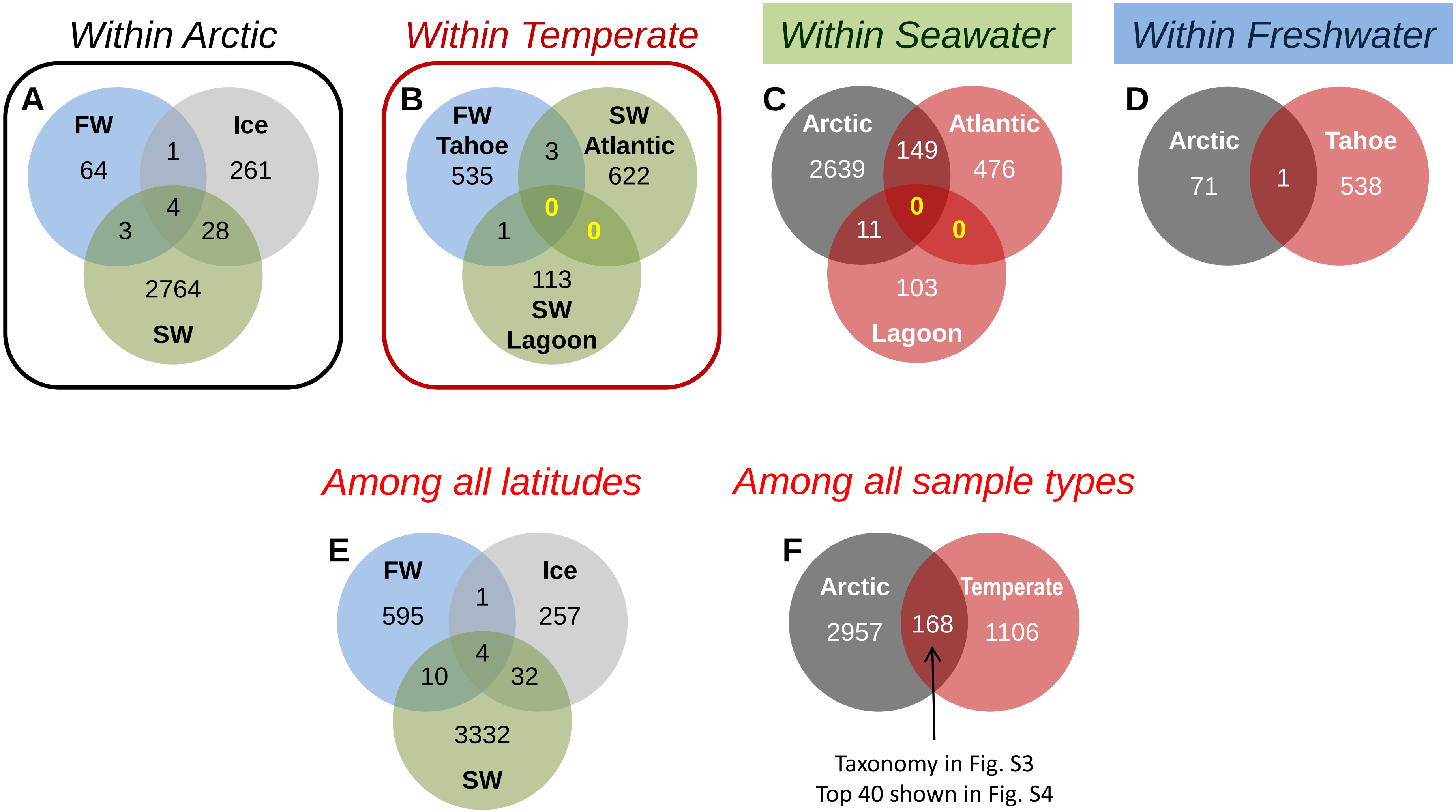

Figure S2. Numbers of shared OTUs (98% identity) between the various sample types. Color-coding for sample types matches Fig. 3 and case numbers (A-F) are referred to in Fig. S3. FW, freshwater; SW, seawater.

		# shared OTUs	Clone CFL161DB09	Crypto-Rozella	Asco-Epicoccum	Asco-Mycochaetophora	Asco-Aureobasidium	Asco-Cladosporium	Asco-Cordyceps	Asco-Engyodontium	Asco-Eurotium	Asco-Meyerozyma	Asco-Mycosphaeralla	Asco-Nectria	Asco-Sarocladium	Basidio-Leucosporidium	Basidio-Sporisorium	Unclassified
**Case A**	Universal FW+Ice+SW	4	1	–	–	–	2	–	–	–	–	–	–	–	–	–	1	–
	Shared FW+Ice	1	–	–	–	–	1	–	–	–	–	–	–	–	–	–	–	–
	Shared Ice+SW	28	24	–	1	–	–	–	–	–	–	–	1	–	1	1	–	–
	Shared SW+FW	3	1	–	–	–	–	–	–	–	–	–	–	–	1	–	–	1
**Case B**	Universal Tahoe+Atlantic+Lagoon	–	–	–	–	–	–	–	–	–	–	–	–	–	–	–	–	–
	Shared Tahoe+Atlantic	3	–	–	–	–	1	1	–	–	–	–	1	–	–	–	–	–
	Shared Atlantic+Lagoon	–	–	–	–	–	–	–	–	–	–	–	–	–	–	–	–	–
	Shared Lagoon+Tahoe	1	–	–	–	–	1	–	–	–	–	–	–	–	–	–	–	–
**Case C**	Universal Arctic+Atlantic+Lagoon	–	–	–	–	–	–	–	–	–	–	–	–	–	–	–	–	–
	Shared Arctic+Atlantic	149	105	11	2	4	5	2	1	2	2	1	1	1	6	2	2	2
	Shared Atlantic+Lagoon	–	–	–	–	–	–	–	–	–	–	–	–	–	–	–	–	–
	Shared Lagoon+Arctic	11	6	–	–	–	3	1	–	–	–	–	–	–	–	1	–	–
**Case D**	Universal Arctic+Tahoe	1	–	–	–	–	–	–	–	–	–	–	–	–	–	–	–	1
**Case E**	Universal FW+Ice+SW	4	1	–	–	–	2	–	–	–	–	–	–	–	–	–	1	–
	Shared FW+Ice	1	–	–	–	–	1	–	–	–	–	–	–	–	–	–	–	–
	Shared Ice+SW	32	27	–	1	1	1	–	–	–	–	–	–	–	1	1	–	–
	Shared SW+FW	10	1	–	–	–	4	1	–	–	–	–	2	–	1	–	–	1
**Case F**	Universal Arctic+Temperate	168	114	11	2	4	11	3	1	2	2	1	2	1	6	3	2	3

Figure S3. Taxonomic identities of the shared OTUs (98% identity) between the various sample types. Color-coding for taxa matches Fig. 3 and case numbers (A-F) refer to Fig. S2. Asco, Ascomycota; Basidio, Basidiomycota; Crypto, Cryptomycota; FW, freshwater; SW, seawater.

Figure S4. Top 40 OTUs. Distribution of sequences within the top 40 shared OTUs (98% identity) showing vastly different profiles between Arctic and Temperate samples.

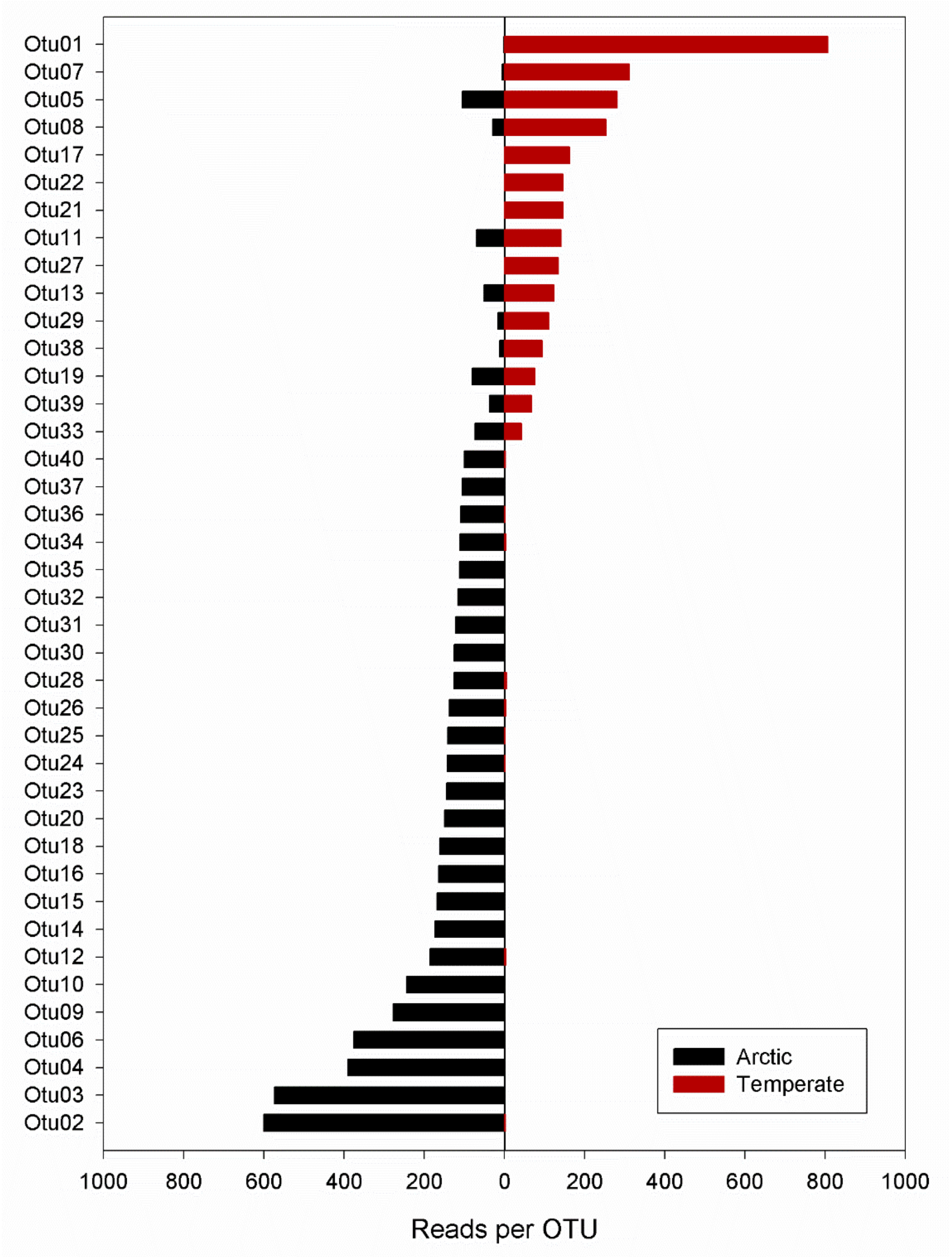

Supplement: Supplementary Figures [file srep30120-s2.pdf]
