# Supplementary material for: Novel chytrid lineages dominate fungal sequences in diverse marine and freshwater habitats
Source: Sci Rep. 2016 Jul 22;6:30120. doi: 10.1038/srep30120 (PMC4957111; doi:10.1038/srep30120)
Supplement: Supplementary Tables [file srep30120-s3.pdf]

Supplementary Information for:

Novel chytrid lineages dominate fungal sequences in diverse marine and freshwater habitats

André M. Comeau, Warwick F. Vincent, Louis Bernier & Connie Lovejoy

Table S1. Characteristics of the arctic and temperate samples sequenced (all projects/sampling date from 2003-2011).

Project (Years)	*Location*[a]	*Salinity*[b]	*# Samples*	*# Stations*	*Depth*[c]	*Size Fraction*[d]	*Molecule*	*Number of pyrosequences* Raw	Post-Filter	Fungi (%)	*Accession*	*Reference*
Arctic Samples												
AMCE (2003-10)	CB/A.Gulf	Marine	11	6	SCM	S	DNA	173,549	108,679	2,791 (2.57)	SRA029114	Comeau et al., 2011
AMELD (2008)	Lake A	FreshW	8	1	M	L+S	DNA	70,330	14,741	49 (0.33)	SRA057195	Charvet et al., 2012
		Marine	4					38,110	7,040	51 (0.72)		
AMELD (2009)	Lake A	FreshW	4	1	M	L	DNA	58,170	29,325	29 (0.10)		
		Marine	1					7,960	3,860	6 (0.16)		
ANITA (2007)	CB	Marine	11	4	M	L	Both	143,779	93,061	1,226 (1.32)	PRJNA217362	Comeau et al.,
ArcticNet (2010)	BaffinB	Marine	1[e]	1	SCM	S	DNA	46,600	28,499	15 (0.05)	PRJNA284115	unpubl.
ArcticNet (2011)	varied	Marine	5	5	SCM	L	DNA	48,242	40,192	113 (0.28)	SRP058096	Luddington et al., submitted
AOS (2005-11)	Arctic Seas	Marine	36	20	M	L+S	DNA	429,492	303,235	2,378 (0.78)	SRP040734	Thaler and Lovejoy, 2015
ASID (2008)	A.Gulf	Ice	12	5	–	L+S	DNA	97,489	21,829	383 (1.75)	SRA054160	Comeau et al., 2013
CFL (2007-8)	A.Gulf	Marine	48	12	Sf	L+S	DNA	126,829	45,296	788 (1.74)	PRJNA283142	Lovejoy, unpubl.
							RNA	126,286	63,927	353 (0.55)		
					SCM		DNA	133,284	35,414	5,369 (15.16)		
							RNA	143,141	76,400	1,723 (2.26)		
Char Lake (2009)	Char Lake	Fresh	1	1	Sf	L	DNA	7,613	4,676	8 (0.17)	SRR2075391	Charvet et al., unpubl.
ICESCAPES (2010)	Ber/Chuk	Marine	23	12	Sf	L	DNA	138,658	64,159	882 (1.37)	SRP029300	Thaler and Lovejoy, 2015
						S		137,727	49,451	263 (0.53)		
MALINA (2009)	CB	Marine	24	6	M	S	DNA	272,260	113,342	417 (0.37)	SRA063446	Monier et al., 2013
Ward Hunt Lake (2009)	WH Isl	Fresh	1	1	Sf	L	DNA	7,534	4,714	1 (0.02)	SRP027540	Charvet et al., 2014

Temperate Samples												
AquaMN MIST (2010)	Magd Isl	Marine	32	6	Sf	L<br>S	RNA	156,033<br>252,979	62,875<br>109,328	5 (<0.01)<br>9 (<0.01)	PRJNA289000<br>PRJNA288997	Péquin, 2015
AquaMN seasonal (2009)	Magd Isl	Marine	10	1	Sf	L+S	DNA	106,797	31,657	118 (0.37)	SRP043016	Péquin et al., submitted
CHONe (2009)	ScotianS	Marine	7	7	SCM	L	DNA	67,168	55,889	4,185 (7.49)	SRP058096	Dasilva et al., 2014
CHONe (2010A)	Gulf of M	Marine	24	6	M	S	DNA<br>RNA	118,407<br>106,041	72,960<br>57,036	26 (0.04)<br>0	SRP040423	Dasilva, 2014
CHONe (2010B)	Gulf of M	Marine	44	4	M	L<br>S	RNA	255,484<br>244,633	55,717<br>82,248	147 (0.26)<br>397 (0.48)		Lovejoy, unpubl.
Lake Tahoe (2011)	CA/NV	Fresh	12	4	M	S	DNA	136,824	81,677	1,531 (1.87)	PRJNA288969	Vincent and Lovejoy, unpubl.
Totals	–	–	319	103	–	–	–	3,651,419	1,717,227	23,263 (1.35)		

[a]A.Gulf, Amundsen Gulf; BaffinB, Baffin Bay; Ber/Chuk, Bering Strait+Chukchi Sea; CA/NV, California/Nevada; CB, Canada Basin (including Beaufort Sea); Gulf of M, Gulf of Maine; Magd Isl, Magdalene Islands; ScotianS, Scotian Shelf; WH Isl, Ward Hunt Island.
[b]FreshW, freshwater.
[c]M, multiple depths; Sf, surface waters (0.5-12 m); SCM, subsurface chlorophyll *a* maximum.
[d]L, large size-fraction (3-50 µm); S, small size-fraction (0.2-3 µm).
[e]This one sample was selected for a GS-FLX+ long-read chemistry test.

References

Charvet, S., Vincent, W.F., Comeau, A.M. & Lovejoy, C. Pyrosequencing analysis of the protist communities in a High Arctic meromictic lake: DNA preservation and change. *Front. Extreme Microbiol.* **3**, 422 (2012).

Charvet, S., Vincent, W.F. & Lovejoy, C. Effects of light and prey availability on Arctic freshwater protist communities examined by high-throughput DNA and RNA sequencing. *FEMS Microbiol. Ecol.* **88**, 550-564 (2014).

Comeau, A.M., Li, W.K.W., Tremblay, J.-E., Carmack, E.C. & Lovejoy, C. Changes in Arctic Ocean microbial community structure following the 2007 record sea ice minimum. *PLoS ONE* **6**, e27492 (2011).

Comeau, A.M. *et al.* Protists in Arctic drift and land-fast sea ice. *J. Phycol.* **49**, 229-240 (2013).

Dasilva, C. Picoplancton de l'Océan Atlantique Nord-Ouest : Diversité et structure des communautés eucaryotes. Université Laval, PhD thesis (2014).

Dasilva, C., Li, W.K.W. & Lovejoy, C. Phylogenetic diversity of eukaryotic marine microbial plankton on the Scotian Shelf Northwestern Atlantic Ocean. *J. Plank. Res.* **36**, 344-363 (2014).

Luddington, I., Lovejoy, C. & Kaczmarska, I. Arctic and Atlantic Ocean Thalassiosirales meta-communities detected using single-cell PCR and high throughput sequencing. *J. Plank. Res.*, under review.

Monier, A. *et al.* Upper Arctic Ocean water masses harbor distinct communities of heterotrophic flagellates. *Biogeosciences* **10**, 4273-4286 (2013).

Péquin, B. Structure et diversité de la communauté de protistes de la lagune de Havre-aux-Maisons, Canada. Université Laval, PhD thesis (2015).

Thaler, M. & Lovejoy, C. Biogeography of heterotrophic flagellate populations indicates the presence of generalist and specialist taxa in the Arctic Ocean. *Appl. Environ. Microbiol.* **81**, 2137-2148 (2015).

Table S2. Taxonomic breakdowns for all groups of arctic and temperate samples sequenced (FW, freshwater; SW, seawater).

								Temperate		
**level**	**rankID**	**taxon**	**lvls**	**total**	**Arc_FW**	**Arc_ICE**	**Arc_SW**	**FW_Tahoe**	**SW_Atl.**	**SW_Lagoon**
0	0	Root	1	23263	87	383	16375	1531	4755	132
1	0.1	Eukaryota	4	23263	87	383	16375	1531	4755	132
2	0.1.8	Fungi/Metazoa	2	23201	87	383	16345	1516	4744	126
3	0.1.8.1	Fungi	10	23119	87	383	16302	1483	4743	121
4	0.1.8.1.2	Chytridiomycota	3	2949	18	1	1772	1131	22	5
5	0.1.8.1.2.1	Chytridiomycetes	10	2929	16	1	1772	1120	15	5
6	0.1.8.1.2.1.1	Chytridiales_sp.PL70	0	121	1	1	19	99	1	0
6	0.1.8.1.2.1.2	Chytridium	0	20	0	0	5	11	3	1
6	0.1.8.1.2.1.3	Cladochytrium	0	2	0	0	0	2	0	0
6	0.1.8.1.2.1.4	Geranomyces	0	13	0	0	0	13	0	0
6	0.1.8.1.2.1.5	Lacustromyces	0	6	0	0	0	6	0	0
6	0.1.8.1.2.1.8	Powellomycetes	0	140	1	0	0	139	0	0
6	0.1.8.1.2.1.9	Rhizophlyctis	0	15	0	0	0	15	0	0
6	0.1.8.1.2.1.10	Rhizophydium	0	2055	4	0	1694	353	0	4
6	0.1.8.1.2.1.11	Spizellomyces	0	176	0	0	0	176	0	0
6	0.1.8.1.2.1.12	unclassified	0	381	10	0	54	306	11	0
5	0.1.8.1.2.2	Monoblepharidomycetes	1	1	0	0	0	1	0	0
6	0.1.8.1.2.2.1	Monoblepharis	0	1	0	0	0	1	0	0
5	0.1.8.1.2.3	unclassified	1	19	2	0	0	10	7	0
6	0.1.8.1.2.3.1	unclassified	0	19	2	0	0	10	7	0
4	0.1.8.1.3	Cryptomycota	1	379	1	0	168	4	204	2
5	0.1.8.1.3.1	Rozella	1	379	1	0	168	4	204	2
6	0.1.8.1.3.1.1	Rozella	0	379	1	0	168	4	204	2
4	0.1.8.1.4	Dikarya(Ascomycota)	9	5439	28	25	4929	56	363	38
5	0.1.8.1.4.3	Dothideomycetes	9	3226	13	22	3061	50	61	19
6	0.1.8.1.4.3.1	Aureobasidium	0	982	4	20	925	14	8	11

6	0.1.8.1.4.3.2	Botryosphaeria	0	1	1	0	0	0	0	0
6	0.1.8.1.4.3.3	Cladosporium	0	57	0	0	30	2	23	2
6	0.1.8.1.4.3.4	Epicoccum	0	2079	2	2	2059	1	13	2
6	0.1.8.1.4.3.5	Leptosphaeria	0	40	4	0	17	6	12	1
6	0.1.8.1.4.3.7	Mycosphaerella	0	29	1	0	3	23	2	0
6	0.1.8.1.4.3.8	Rhytidhysteron	0	1	0	0	0	0	0	1
6	0.1.8.1.4.3.9	Teratosphaeria	0	6	0	0	6	0	0	0
6	0.1.8.1.4.3.10	unclassified	0	31	1	0	21	4	3	2
5	0.1.8.1.4.4	Eurotiomycetes	3	77	4	1	50	0	4	18
6	0.1.8.1.4.4.5	Eurotium	0	61	4	1	49	0	4	3
6	0.1.8.1.4.4.6	Talaromyces	0	15	0	0	0	0	0	15
6	0.1.8.1.4.4.7	unclassified	0	1	0	0	1	0	0	0
5	0.1.8.1.4.8	Leotiomycetes	2	7	5	0	2	0	0	0
6	0.1.8.1.4.8.1	Blumeria	0	1	0	0	1	0	0	0
6	0.1.8.1.4.8.2	Botryotinia	0	6	5	0	1	0	0	0
5	0.1.8.1.4.11	Orbiliomycetes	1	1	1	0	0	0	0	0
6	0.1.8.1.4.11.1	Orbilia	0	1	1	0	0	0	0	0
5	0.1.8.1.4.12	Pezizomycetes	1	1	0	0	1	0	0	0
6	0.1.8.1.4.12.1	Tuber	0	1	0	0	1	0	0	0
5	0.1.8.1.4.14	Saccharomycetes	4	21	2	0	10	3	6	0
6	0.1.8.1.4.14.1	Candida	0	1	1	0	0	0	0	0
6	0.1.8.1.4.14.2	Kluyveromyces	0	1	0	0	1	0	0	0
6	0.1.8.1.4.14.4	Meyerozyma	0	17	0	0	8	3	6	0
6	0.1.8.1.4.14.6	Saccharomyces	0	2	1	0	1	0	0	0
5	0.1.8.1.4.16	Sordariomycetes	6	335	1	1	62	0	271	0
6	0.1.8.1.4.16.5	Cordyceps	0	47	0	0	11	0	36	0
6	0.1.8.1.4.16.6	Isaria	0	4	0	0	0	0	4	0
6	0.1.8.1.4.16.8	Nectria	0	4	0	0	4	0	0	0
6	0.1.8.1.4.16.9	Neurospora	0	7	0	0	0	0	7	0
6	0.1.8.1.4.16.11	Sarocladium	0	267	1	1	44	0	221	0
6	0.1.8.1.4.16.13	unclassified	0	6	0	0	3	0	3	0

5	0.1.8.1.4.18	mitosporic_Ascomycota	2	1745	0	1	1722	0	21	1
6	0.1.8.1.4.18.1	Engyodontium	0	104	0	0	87	0	16	1
6	0.1.8.1.4.18.2	Mycochaetophora	0	1641	0	1	1635	0	5	0
5	0.1.8.1.4.19	unclassified	1	26	2	0	21	3	0	0
6	0.1.8.1.4.19.1	unclassified	0	26	2	0	21	3	0	0
4	0.1.8.1.5	Dikarya(Basidiomycota)	4	446	12	2	378	0	50	4
5	0.1.8.1.5.5	Cystobasidiomycetes	1	4	0	0	4	0	0	0
6	0.1.8.1.5.5.1	Cystobasidium	0	4	0	0	4	0	0	0
5	0.1.8.1.5.7	Exobasidiomycetes	2	8	0	0	8	0	0	0
6	0.1.8.1.5.7.1	Entyloma	0	1	0	0	1	0	0	0
6	0.1.8.1.5.7.3	Malassezia	0	7	0	0	7	0	0	0
5	0.1.8.1.5.8	Microbotryomycetes	1	284	11	1	222	0	48	2
6	0.1.8.1.5.8.1	Leucosporidium	0	284	11	1	222	0	48	2
5	0.1.8.1.5.13	Ustilaginomycetes	2	150	1	1	144	0	2	2
6	0.1.8.1.5.13.2	Sporisorium	0	146	1	1	140	0	2	2
6	0.1.8.1.5.13.5	unclassified	0	4	0	0	4	0	0	0
4	0.1.8.1.6	Entomophthoromycota	1	1	0	0	1	0	0	0
5	0.1.8.1.6.1	Entomophthoromycotina	1	1	0	0	1	0	0	0
6	0.1.8.1.6.1.1	Entomophthora	0	1	0	0	1	0	0	0
4	0.1.8.1.8	Incertae_sedis(ArcticBasalClones)	1	13477	17	354	9010	0	4038	58
5	0.1.8.1.8.1	ArcticBasalClones	1	13477	17	354	9010	0	4038	58
6	0.1.8.1.8.1.1	clone_CFL161DB09	0	13477	17	354	9010	0	4038	58
4	0.1.8.1.11	Incertae_sedis(Mucoromycotina)	1	1	0	0	1	0	0	0
5	0.1.8.1.11.1	Mucoromycotina	1	1	0	0	1	0	0	0
6	0.1.8.1.11.1.2	Mucor	0	1	0	0	1	0	0	0
4	0.1.8.1.12	Incertae_sedis(Nephridiophagidae)	1	1	0	0	0	1	0	0
5	0.1.8.1.12.1	Nephridiophagidae	1	1	0	0	0	1	0	0
6	0.1.8.1.12.1.1	Nephridiophaga	0	1	0	0	0	1	0	0
4	0.1.8.1.13	Incertae_sedis(Olpidiaceae)	1	1	0	0	0	1	0	0
5	0.1.8.1.13.1	Olpidiaceae	1	1	0	0	0	1	0	0
6	0.1.8.1.13.1.1	Olpidium	0	1	0	0	0	1	0	0

4	0.1.8.1.17	unclassified	1	425	11	1	43	290	66	14
5	0.1.8.1.17.1	unclassified	1	425	11	1	43	290	66	14
6	0.1.8.1.17.1.1	unclassified	0	425	11	1	43	290	66	14
3	0.1.8.3	unclassified	1	82	0	0	43	33	1	5
4	0.1.8.3.1	unclassified	1	82	0	0	43	33	1	5
5	0.1.8.3.1.1	unclassified	1	82	0	0	43	33	1	5
6	0.1.8.3.1.1.1	unclassified	0	82	0	0	43	33	1	5
2	0.1.22	unclassified	1	57	0	0	27	15	10	5
3	0.1.22.1	unclassified	1	57	0	0	27	15	10	5
4	0.1.22.1.1	unclassified	1	57	0	0	27	15	10	5
5	0.1.22.1.1.1	unclassified	1	57	0	0	27	15	10	5
6	0.1.22.1.1.1.1	unclassified	0	57	0	0	27	15	10	5
